# Structure modulation of helix 69 from *Escherichia coli* 23S ribosomal RNA by pseudouridylations

**DOI:** 10.1093/nar/gkt1329

**Published:** 2013-12-26

**Authors:** Jun Jiang, Raviprasad Aduri, Christine S. Chow, John SantaLucia

**Affiliations:** Department of Chemistry, Wayne State University, Detroit, MI 48202, USA

## Abstract

Helix 69 (H69) is a 19-nt stem-loop region from the large subunit ribosomal RNA. Three pseudouridine (Ψ) modifications clustered in H69 are conserved across phylogeny and known to affect ribosome function. To explore the effects of Ψ on the conformations of *Escherichia coli* H69 in solution, nuclear magnetic resonance spectroscopy was used to reveal the structural differences between H69 with (ΨΨΨ) and without (UUU) Ψ modifications. Comparison of the two structures shows that H69 ΨΨΨ has the following unique features: (i) the loop region is closed by a Watson–Crick base pair between Ψ1911 and A1919, which is potentially reinforced by interactions involving Ψ1911N1H and (ii) Ψ modifications at loop residues 1915 and 1917 promote base stacking from Ψ1915 to A1918. In contrast, the H69 UUU loop region, which lacks Ψ modifications, is less organized. Structure modulation by Ψ leads to alteration in conformational behavior of the 5' half of the H69 loop region, observed as broadening of C1914 non-exchangeable base proton resonances in the H69 ΨΨΨ nuclear magnetic resonance spectra, and plays an important biological role in establishing the ribosomal intersubunit bridge B2a and mediating translational fidelity.

## INTRODUCTION

**Figure 1.**
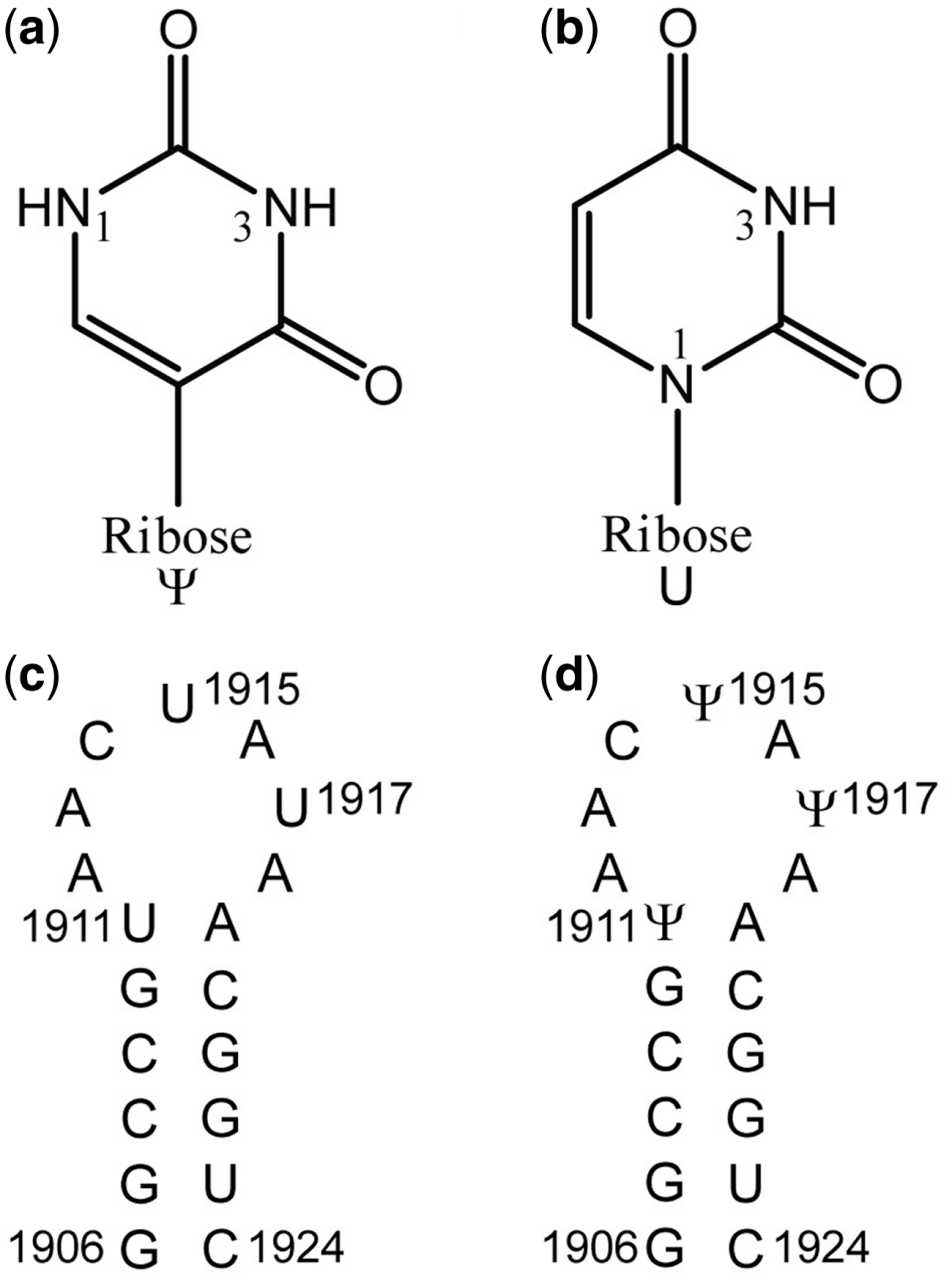
Sequence and modification sites of H69 from *E. coli* 23S rRNA are shown. (**a**) A pseudouridine (Ψ) has a C5–C1' glycosidic bond. (**b**) A uridine residue contains an N1–C1' glycosidic bond. The numbering for the imino protons of U and Ψ is given. (**c**) The RNA construct without pseudouridylation, UUU, is given with the *E. coli* numbering. (**d**) The ΨΨΨ construct shows pseudouridine (Ψ) residues at positions 1911, 1915 and 1917.

RNA molecules can adopt highly folded 3D structures to carry out their essential structural and catalytic functions in biological systems (1). As enrichment to the four standard nucleotides (i.e*.* A, C, G and U), post-transcriptional modifications enhance the chemical repertoire of RNA and play important roles in ‘fine-tuning’ local conformations of RNA (2,3). Among the >100 modifications identified to date (4), pseudouridine (Ψ) (Figure 1a) was the first reported and is also the most frequently encountered (5). Uridine (Figure 1b) is isomerized to Ψ (Figure 1a) by replacing the N-glycosidic bond with a C-glycosidic bond, and this covalent structural variation has been shown to modulate local conformation and overall activity in telomerase (6), spliceosomal (7) and transfer (8) RNAs. In the *Escherichia coli* (*E. coli*) ribosomal RNAs (rRNAs), 10 Ψ modifications are distributed in the functionally important regions (e.g*.* the peptidyl transferase center (PTC) and the intersubunit bridge B2a) (9,10), with the latter hosting three Ψs in a 19-nt-long hairpin segment of the 23S rRNA named helix 69 (H69).

Helix 69 shows a high degree of conservation in both sequence and secondary structure across phylogeny (11). An additional conserved feature of H69 is the existence of multiple pseudouridylation sites (*E. coli* numbering, positions 1911, 1915 and 1917; Figure 1c and d), which have been mapped in *E. coli*, human and several other organisms (9,10,12–14). Helix 69 plays a central role, together with helix 44 (h44) of the small subunit rRNA (16S and 16S-like), in establishing the B2a intersubunit bridge, one of the most highly conserved regions of the ribosome (15–19), and has direct contacts with multiple factors during the translation process, as revealed in crystal and cryo-EM structures (18,20–26). Together, these facts point to the significance of the conserved Ψs in folding and function of H69 (27,28).

Mutagenesis studies provided insight into the conservation of sequence and/or post-transcriptional modifications in H69. Deletion of H69 in bacterial ribosomes leads to the disruption of the B2a intersubunit bridge, which in turn results in defects in ribosome association and peptide release (29). Single-nucleotide mutations in H69 confer slow-growth phenotypes with compromised protein synthesis activity and translational fidelity (30–33). Among the H69 loop residues most sensitive to single-nucleotide mutations, Ψ1915, A1916 and Ψ1917 show reduced growth rates and interrupted ribosome assembly (33). It was revealed in the post-transcriptional modification mapping of H69 that the mutation A1916G reduces isomerization of U1917 to Ψ1917, and A1916U abolishes the modification activity completely (34). Therefore, the slow-growth phenotype observed with A1916G is partially attributed to reduced ribosome assembly due to lowered Ψ1917 modification levels. A growth advantage with pseudouridylation at positions 2258 and 2260 in *Saccharomyces cerevisiae* (corresponding to 1915 and 1917 in *E. coli*) in the H69 loop was also reported (35).

Thermodynamic and chemical-probing studies were carried out previously to explore the effects of pseudouridylation on H69. It was demonstrated on other RNA motifs that Ψ can stabilize RNA folding by formation of water-mediated hydrogen-bonding interactions through ΨN1H (36); however, a stabilizing effect was only observed at position Ψ1911 in H69 model oligonucleotide hairpins from *E. coli* and human. The loop-closing Ψ contributed −0.6 to −1.1 kcal/mol to the *ΔG°_37_* of the RNA stem-loop structure (37,38). In contrast, Ψ1915 and Ψ1917, individually or collectively, showed slight destabilizing effects in the same model studies (37,38). Corresponding differences in H69 flexibility were observed through SHAPE (39) analysis of 50S subunits from *E. coli* wild-type and RluD^−^ (Ψ-deficient) strains; only A1913 and A1918 in the wild-type 23S rRNA showed strong reactivity toward the SHAPE reagent, whereas all H69 loop residues demonstrated mild reactivity in the unmodified RNA (RluD^−^ 23S rRNA) (40).

To elucidate in more detail the structural effects of Ψ modifications on H69 folding and explore correlations between the modifications and their biological significance, the solution structures of RNA constructs with (ΨΨΨ) and without (UUU) pseudouridylations (Figure 1c and d), representing H69 from *E. coli* 23S rRNA, were examined by using nuclear magnetic resonance (NMR) spectroscopy. A comparison of the two structures reveals that Ψs substantially alter the folding of the H69 loop region. In UUU, the base moieties of all three loop U residues are found to have greater solvent accessibility than the corresponding Ψ residues in ΨΨΨ, which may help with RluD recognition and catalysis. The Ψ1911 forms a Watson–Crick base pair with A1919 and has unique hydrogen-bonding interactions. The NMR structure of ΨΨΨ also shows that Ψ1915 and Ψ1917 participate in base stacking in the 3' half of the H69 loop. Together, the three Ψ modifications influence conformational behavior of the 5' half of the H69 loop region, as shown by line-width broadening of the C1914 base non-exchangeable protons, and are suggested to play a role in facilitating base flipping of A1913, which is known to make important contacts in the B2a intersubunit bridge of intact ribosomes (41).

## MATERIALS AND METHODS

### Preparation of H69 RNA oligonucleotides

Unmodified H69 RNA samples (UUU, 5'-GGCCGUAACUAUAACGGUC-3') were synthesized by *in vitro* T7 RNA polymerase transcription with unlabeled or ^13^C, ^15^N-labeled NTPs, synthetic gel-purified DNA template, and promoter sequences (42). Full-length H69 RNA transcripts were purified by using denaturing 20% (w/v) preparative polyacrylamide gel electrophoresis and electroelution in 0.2 × Tris borate + ethylenediaminetetraacetic acid buffer with a Schleicher and Schuell® Elutrap. RNAs were desalted with Sep-pak® (Waters) reverse-phase chromatography cartridges, and the eluted fractions were pooled and lyophilized to a powder.

Synthetic modified RNA (37) (ΨΨΨ, 5'-GGCCGΨAACΨAΨAACGGUC-3') was purchased from Dharmacon® (Thermo Scientific) and subjected to high-performance liquid chromatography purification on a Waters Xterra MS C18 column. A gradient of acetonitrile from 6.0 to 7.8% over 24 min in 25 mM of triethylammonium acetate, pH 6.5, at a flow rate of 3 ml/min was used. The RNA-containing fractions were lyophilized and desalted with a Sep-Pak column. The molecular masses of the RNA oligonucleotides were confirmed by using MALDI-TOF mass spectrometry.

### Preparation of RNA NMR samples

Purified H69 oligonucleotides (UUU and ΨΨΨ) were dissolved in 300 μl of 10 mM potassium phosphate, 50 mM KCl, pH 7.3, and 0.1 mM Na_2_-ethylenediaminetetraacetic acid. The samples were lyophilized to a powder. Deuterium oxide (D_2_O, 99.96%; Cambridge Isotope Labs) was used to exchange the residual H_2_O twice by lyophilization, and the samples were dissolved to a final volume of 300 μl for NMR experiments on non-exchangeable protons. A H_2_O/D_2_O (90%/10%) mixture (300 µl) was used for experiments on exchangeable protons. All of the NMR samples contained ∼1.0 mM RNA and a trace amount of 3-(trimethylsilyl) propionate (TSP) as an internal proton chemical shift reference.

### NMR spectroscopy

All NMR experiments were carried out on a Bruker Avance 700 MHz spectrometer equipped with an HCN cryoprobe and a Varian Mercury 400 MHz equipped with a room temperature QXI probe for ^31^P NMR experiments. Spectra for exchangeable proton resonance assignments and base-pairing identification were acquired at 283 and 288 K. All other spectra of UUU and ΨΨΨ samples were recorded at 298 and 310 K, respectively. Topspin 2.1 (Bruker) and Sparky 3.114 (University of California, San Francisco, CA, USA) were used for spectral processing and analysis.

The 2D NOESY spectra of the unlabeled H69 RNA samples (UUU and ΨΨΨ) dissolved in 99.96% D_2_O were initially analyzed to assign the resonances of base protons (H8/H6) and sugar protons (H1'). Standard NMR experiments (e.g. 2D NOESY, 2D DQF-COSY, 2D ^13^C-^1^H HMQC and 3D TOCSY-NOESY) were collected on unlabeled samples, whereas 2D ^13^C-^1^H CT-HSQC, 3D HCcH-COSY, 3D HCcH-TOCSY, 3D HCCh-TOCSY and 3D NOESY-HMQC were run on the ^13^C,^15^N-labeled sample (for UUU only) to assign H/C resonances. The 1D ^31^P and 2D ^31^P-^1^H HETCOR experiments on unlabeled constructs were used to identify chemical shifts of ^31^P resonances (UUU and ΨΨΨ).

### Structure calculations

Interproton distance restraints were derived from 2D NOESY spectra as described previously (43). Interproton distance restraints of exchangeable protons were only applied to residues involved in base pairs in the stem region (from G1906–C1924 to G1910–C1920 for UUU and from G1906–C1924 to Ψ1911–A1919 for ΨΨΨ) (44).

The dihedral angles, α, β, γ, δ, ε, ζ and χ, of residues in the stem region (from G1906–C1924 to G1910–C1920 for UUU and from G1906–C1924 to Ψ1911–A1919 for ΨΨΨ) were restrained according to the A-form RNA helix geometry (−62 ± 10°, 172 ± 10°, 60 ± 10°, 84 ± 10°, −160 ± 10°, −71 ± 10°, and −160 ± 10°, respectively). Loop residue dihedral angles α and ζ were restrained (0 ± 120°) to exclude the *trans* conformation, owing to absence of any downfield-shifted ^31^P resonances in UUU and ΨΨΨ 1D ^31^P spectra (45,46). Because only weak cross-peaks between P-H5' or P-H5” were observed, the β-dihedral angles of loop residues were loosely restrained to −180 ± 90°, which essentially covers the entire sterically accessible range (47). The γ dihedral angles were restrained to be 120 ± 120° to exclude *gauche^-^* conformations which are rarely observed. Cross-peaks from J-coupling of H1' and H2' in the 2D DQF-COSY were used to determine the δ dihedral angles of loop residues, in which an intense cross-peak comparable with the pyrimidine H6–H5 cross-peaks indicates a C2'-*endo* conformation, and the δ dihedral angle was restrained to 157 ± 40°. If the sugar H1'–H2' cross-peak was weak or invisible, then the δ dihedral angle was restrained to 84 ± 20° corresponding to C3'-*endo* conformation; otherwise, it was left unrestrained (48,49). The ε dihedral angles of loop residues were restrained to be −120 ± 120° to exclude the *gauche^+^* conformation. Owing to the absence of intense cross-peaks between base protons H8/H6 and sugar protons H1' in the 2D NOESY spectra with a short mixing time (τ_m_ = 60 ms) from UUU and ΨΨΨ, the χ dihedral angles of loop residues were all restrained to −110 ± 110° to exclude a *syn* conformation.

The Crystallography and NMR System (CNS) version 1.2 using a simulated annealing and restrained molecular dynamics protocol was used in structure calculations (50). Parameterization of Ψ residues was used as previously reported (51). An extended structure containing only the RNA sequence and covalent linkages of the RNA construct generated using the CNS 1.2 was subjected to a torsion angle molecular dynamics at 20 000 K for 40 000 steps (1 fs/step, 40 ps in total) with molecular dynamics force constants of 50 kcal/mol/Å^2^ and 150 kcal/mol/rad^2^ for the distance restraints and dihedral angle restraints, respectively. Molecular dynamics pseudopotential force constants of 100 kcal/mol/Å^2^ and 250 kcal/mol/rad^2^ were then applied to distance and dihedral restraints, respectively, in the first round (50 000 steps in 100 ps) of slow cooling down to 0 K. In the second round (10 000 steps in 35 ps) of slow cooling down from 2000 to 0 K, a Cartesian molecular dynamics simulation was used (using pseudopotential force constants of 200 kcal/mol/Å^2^ for the distance restraints and 500 kcal/mol/rad^2^ for the dihedral restraints). A force constant of 50 kcal/mol/Å^2^ was used for the base pair planarity restraints in the final energy minimization stage consisting of 800 steps for 20 rounds (using pseudopotential force constants of 300 kcal/mol/Å^2^ for the distance restraints and 700 kcal/mol/rad^2^ for the dihedral restraints). Qualified output structures (i.e. those structures with no distance violation >0.3 Å or dihedral violation >5°) were chosen for further torsion angle molecular dynamics refinement with an energy penalty of 5 kcal/mol equivalent to an NOE violation of 0.3 Å or a dihedral angle violation of 5°. RNA123 (DNA Software, Inc.) was used for restraint violation analysis in NMR structure calculations and refinement for all pair-wise root-mean-square deviation (RMSD) calculations in this work (52). Structure calculation restraints and statistics are shown in Table 1. All the structures were visualized with Pymol (The PyMol Molecular Graphics System, Schrödinger, LLC.).

**Table 1.** Structure calculation restraints and statistics of UUU and ΨΨΨ

Restraints	UUU	ΨΨΨ
NOE distance restraints	241	224
Intra-residue	97	81
Inter-residue	144	143
Stem constraints	59	59
Base pair planarity	5	5
Dihedral angle restraints	128	127
Statistics		
Violations		
NOE distance violation >0.3 Å	0	0
Dihedral angle violation >5°	0	0
Average pair-wise RMSD (Å) of all atoms (10 structures)	1.15	0.66

## RESULTS AND DISCUSSION

### NMR spectroscopy of H69 UUU and ΨΨΨ

The oligonucleotides used in this study correspond to nucleotides 1906–1924 in *E. coli* 23S rRNA, either without (UUU) or with (ΨΨΨ) pseudouridine modifications at positions 1911, 1915 and 1917 (Figure 1c and d). In addition to a natural abundance UUU sample, a ^13^C,^15^N-labeled sample of UUU was synthesized by *in vitro* transcription catalyzed by T7 polymerase and used for assignments of overlapping resonances. The structure families of the two oligonucleotides show comparable quality and convergence (Table 1). The final solution structures of UUU and ΨΨΨ are consistent with all of the observed NMR data, and support the results of previous structural, biophysical and chemical-probing studies besides providing further information on the structural role played by the Ψs in the formation of the intersubunit bridge B2a.

**Figure 2.**
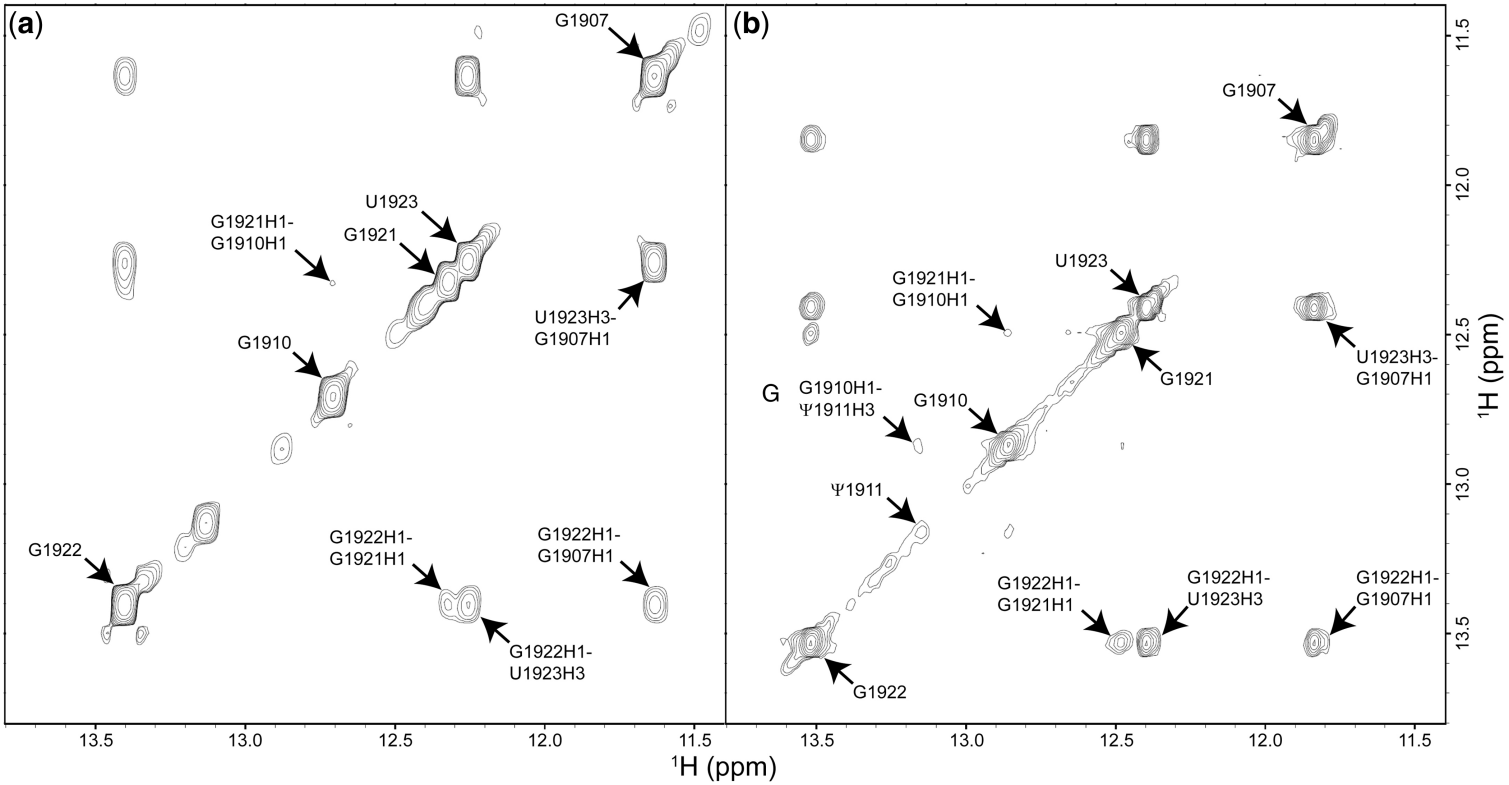
2D NOESY spectra of UUU (**a**) and ΨΨΨ (**b**) dissolved in H_2_O/D_2_O (90/10%). Assignments of the base imino protons and cross-peaks of imino proton pairs are indicated.

Resonances of imino protons from the stem residues of both oligonucleotides were observed in the 2D NOESY spectra (H_2_O/D_2_O: 90/10%; Figure 2) (53). Cross-peaks of the two imino protons from the neighboring base pairs were shown to have medium to weak intensity. The chemical shifts of G1907N1H and U1923N3H are upfield shifted relative to the other imino protons, and an intense cross-peak of the two protons exists for both UUU and ΨΨΨ, consistent with a wobble G•U mismatch structure. Formation of this G•U wobble pair protects the G1907N1H and U1923N3H from being exchanged with solvent; therefore, the corresponding resonances are clearly visible in the 2D NOESY spectra (Figure 2). The assignment of the Ψ1911N3H resonance was confirmed based on cross-peaks with G1910N1H and A1919H2 (Supplementary Figure S1a). The strong Ψ1911N3H to A1919H2 cross-peak is consistent with the formation of Watson–Crick hydrogen bonding. One upfield-shifted imino proton resonance at 10.2 ppm was observed in the 1D imino proton spectrum of ΨΨΨ, and this proton is positioned in proximity to Ψ1911H6 by an intense cross-peak shown in the 2D NOESY (H_2_O/D_2_O: 90/10%) spectrum (Supplementary Figure S1b). This resonance is unambiguously assigned as Ψ1911N1H and its chemical shift value is in good agreement with previous studies (53). No resonances from other Ψ residues in the loop region were observed, suggesting that they are exposed to solvent.

The H8/6 to H1' ‘walk’ pattern of the UUU stem region in the 2D NOESY (D_2_O: 99.96%) spectrum corresponds well with that of ΨΨΨ (Supplementary Figure S2a and b). The chemical shifts of H1's of the three Ψ residues in ΨΨΨ are upfield shifted compared with those of the corresponding uridine residues in UUU by 0.9, 1.1 and 1.3 ppm, respectively, consistent with values reported in other Ψ-containing RNAs (6,54–56). The upfield shift of H1' in Ψ compared with U can be explained by the fact that the less electronegative C5 of Ψ replaces N1 of U at the glycosidic bond position (57,58). Protons U/Ψ1911H6, A1916H2, A1916H1', U/Ψ1917H6 and A1918H2 also show chemical-shift differences of >0.1 ppm (Supplementary Figure S3) between the two constructs. In the 2D NOESY (D_2_O: 99.96%) spectrum of ΨΨΨ, the line width of the C1914 H6–H5 cross-peak is significantly broadened compared with that of UUU (Supplementary Figure S2c and d).

Additional dihedral angle restraints were obtained from 2D DQF-COSY spectra. In the 2D DQF-COSY spectrum of UUU, three intense cross-peaks of H1'–H2' were assigned to C1914, U1915 and U1917 (Supplementary Figure S4a), indicating a C2'-*endo* sugar pucker conformation. In the 2D DQF-COSY of the ΨΨΨ sample, no H1'–H2' cross-peaks are observed. However, the ΨΨΨ spectrum does have one upfield-shifted cross-peak of weak intensity corresponding to H6–H1' of Ψ1915 (Supplementary Figure S4b). The appearance of this *^4^J_HH_-coupling* enables the addition of a dihedral angle (H1'-C1'-C5-C6) constraint of 180 ± 45°, as a coplanar geometry of H6-C6-C5-C1'-H1' is required to maximize the four-bond magnetization transfer, and no strong cross-peak between the H6 and H1' was observed in the corresponding 2D NOESY (D_2_O: 99.96%) spectrum, excluding the possibility of a *syn^χ^* dihedral angle.

### Solution structures of UUU and ΨΨΨ

**Figure 3.**
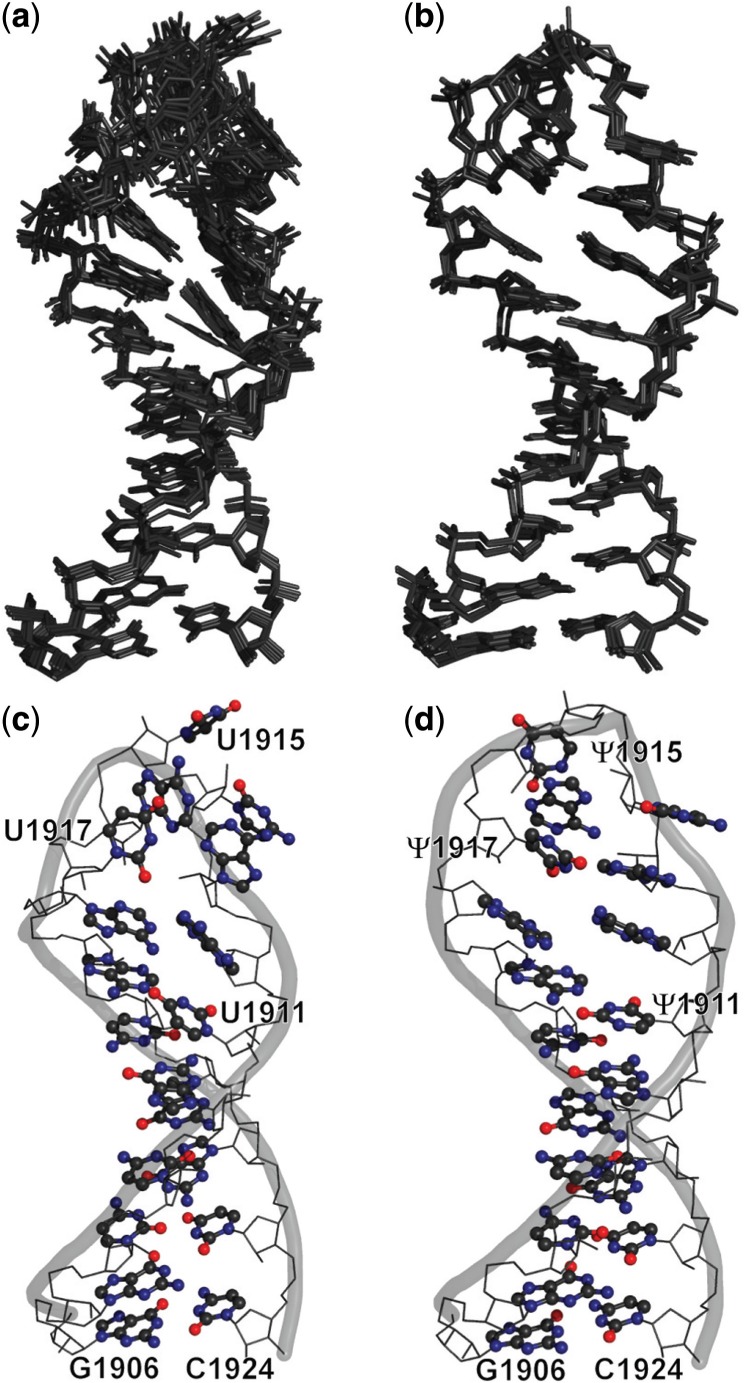
NMR solution structure families (**a** and **b**) and the lowest energy structures (**c** and **d**) of UUU (a and c) and ΨΨΨ (b and d) are shown. In each of the structure families, 10 of the converged lowest energy structures were superimposed by ‘alignment’ with PyMol (The PyMol Molecular Graphics System, Schrödinger, LLC.). The residues subjected to Ψ modifications are labeled in (c and d). The backbone trace is shown as a gray ribbon, the base moieties are shown in ball and stick representation and the remaining are shown as a wire frame.

A total of 241 and 224 NOE restraints were used in the structure calculations of UUU and ΨΨΨ, respectively (Table 1). Ten converged lowest-energy structures from each oligonucleotide were superimposed. An averaged pair-wise all-atom RMSD within the structure family of UUU is 1.15 Å and that of ΨΨΨ is 0.66 Å (Figure 3a and b). In these structures, UUU and ΨΨΨ are both folded into stem-loop structures. To compare the structural effects of Ψ modifications, the lowest-energy structures of UUU and ΨΨΨ were chosen to represent the NMR solution structures of the two oligonucleotides (Figure 3c and d).

#### UUU and ΨΨΨ stem regions are folded into A-form duplexes containing a G•U wobble pair

The stem regions (i.e. residues G1906–G1910 and C1920–C1924) of UUU and ΨΨΨ are folded into an A-form duplex conformation (Figure 3), similar to that of H69 in X-ray crystal structures 1NKW (isolated large ribosomal subunit from *Deinococcus radiodurans* with the same sequence and Ψ modifications of H69 as *E. coli*) and 2I2T (large ribosomal subunit from *E. coli* assembled in complete ribosomes) (59,60). In the crystal structures of H69 from both bacteria and eukaryotes, G1907 and U1923 (*E. coli* numbering) form a wobble pair with two hydrogen bonds (G1907O6–U1923N3H and G1907N1H–U1923O2) (17,59-61), and this structural feature was also observed in the solution structures of UUU and ΨΨΨ (Supplementary Figure S5). The interproton distances between the imino proton of G1922 and the imino protons of G1907 and U1923 (Supplementary Figure S5) support an A-form RNA base-stacking arrangement involving the G1907•U1923 wobble pair (44).

#### Ψ1911 and A1919 form a canonical Watson–Crick base pair with an additional intraresidue hydrogen bond involving Ψ1911N1H

**Figure 4.**
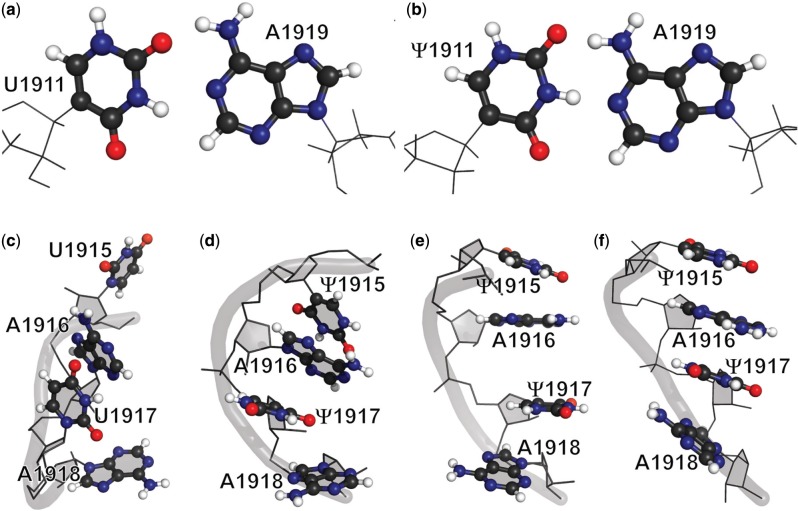
Local conformations of structural motifs involved in Ψ modification(s) in NMR structures and crystal structures are shown. Unlike U1911 and A1919 in UUU (**a**), Ψ1911 and A1919 in ΨΨΨ (**b**) are positioned to form an optimal Watson–Crick base pair. In ΨΨΨ, base moieties of residues from Ψ1915 to A1918 form a continuous-stacking conformation (**d**), which is not observed in the same region of UUU (**c**). This continuous base stacking is also observed in the crystal structures of *D. radiodurans* (PDB ID 1NKW) (**e**) and *E. coli* (PDB ID 2I2T) (**f**) ribosomes.

Neighboring the stem-closing base pair (G1910-C1920), U1911 and A1919 in UUU do not form an optimal Watson–Crick base pair (Figure 4a). In the family of 10 UUU NMR structures, the average distances between hydrogen-bond partner pairs UN3H–AN1 and UO4–AN6H are both 2.5 Å (2.5 ± 0.3 Å and 2.5 ± 0.6 Å, respectively), whereas the optimal hydrogen-bond distance is 1.8 Å. These longer hydrogen-bond distances may reflect dynamics in the U1911–A1919 pair or a paucity of NMR restraints. The sharp line widths for non-exchangeable resonances at these positions suggest that the distorted U–A geometry is due to a lack of NMR restraints. However, it is worth noting that the H69 construct with U1911 is 1.0 kcal/mol less stable than a construct with Ψ1911 (37). In contrast, Ψ1911 and A1919 in ΨΨΨ form a canonical Watson–Crick base pair (Figure 4b). The average distances of ΨN3H–AN1 and ΨO2–AN6H are 1.8 ± 0.1 Å and 1.7 ± 0.2 Å, respectively, and the corresponding three-atom geometry is much closer to a linear arrangement (170 ± 7° for ΨN3-ΨN3H-AN1 and 164 ± 6° for ΨO2-AN6H-AN6). Formation of the Ψ1911–A1919 base pair excludes solvent from the Watson–Crick edge of the two bases, leading to protection of these imino groups from CMCT and DMS chemical probing in the wild-type pseudouridylated 50S ribosomal subunit (40,62). Pseudouridine modification has also been shown to promote the formation of an optimal Watson–Crick base pair in tRNA^Tyr^ from *Bacillus subtilis* to lock down the stem-loop transition and alter the conformation of the neighboring C•A^+^ base pair on the loop side (55).

The local structural rigidity of the stem-loop transition region of H69 may be further enhanced by potential water-mediated hydrogen-bonding interactions involving Ψ1911N1H (36). Even though Ψ1911N1H is exposed to the solvent (Supplementary Figure S6) and no partner is readily available within the direct hydrogen-bonding distance, the presence of a relatively sharp Ψ1911N1H resonance in the imino–proton spectrum suggests that this proton is protected from solvent exchange by a hydrogen bond, possibly mediated by a water molecule (2,36,58). In the family of 10 ΨΨΨ solution structures, the Ψ1911N1H-O2P distance is 4.1 ± 0.1 Å and the N1-N1H-O2P angle is 115 ± 1° (Supplementary Figure S7a). Formation of a hydrogen bond involving Ψ1911N1H, together with base pairing of Ψ1911 and A1919, may explain the large chemical shift difference observed between the H6s of U1911 and Ψ1911 (Supplementary Figure S3). A similar local geometry has been observed in other pseudouridylated RNAs (6) and the crystal structures of H69 in the isolated large subunit and 70S ribosomes (PDB IDs 1NKW and 2I2T, Supplementary Figure S7b and c) (59,60). A thermodynamic stabilizing effect of Ψ modification at 1911 has also been reported, in the context of a complete H69 hairpin (−1.0 kcal/mol) (37) and double-stranded constructs mimicking the stem region (residues from G1906 to A1912 and A1919–C1924 of H69, −1.1 kcal/mol) (38). These values agree with a thermodynamic contribution of −0.7 kcal/mol in conformational stability resulting from a water-mediated hydrogen bond to ΨN1H (36,58,63,64).

#### Ψ1915 and Ψ1917 promote local base stacking

**Figure 5.**
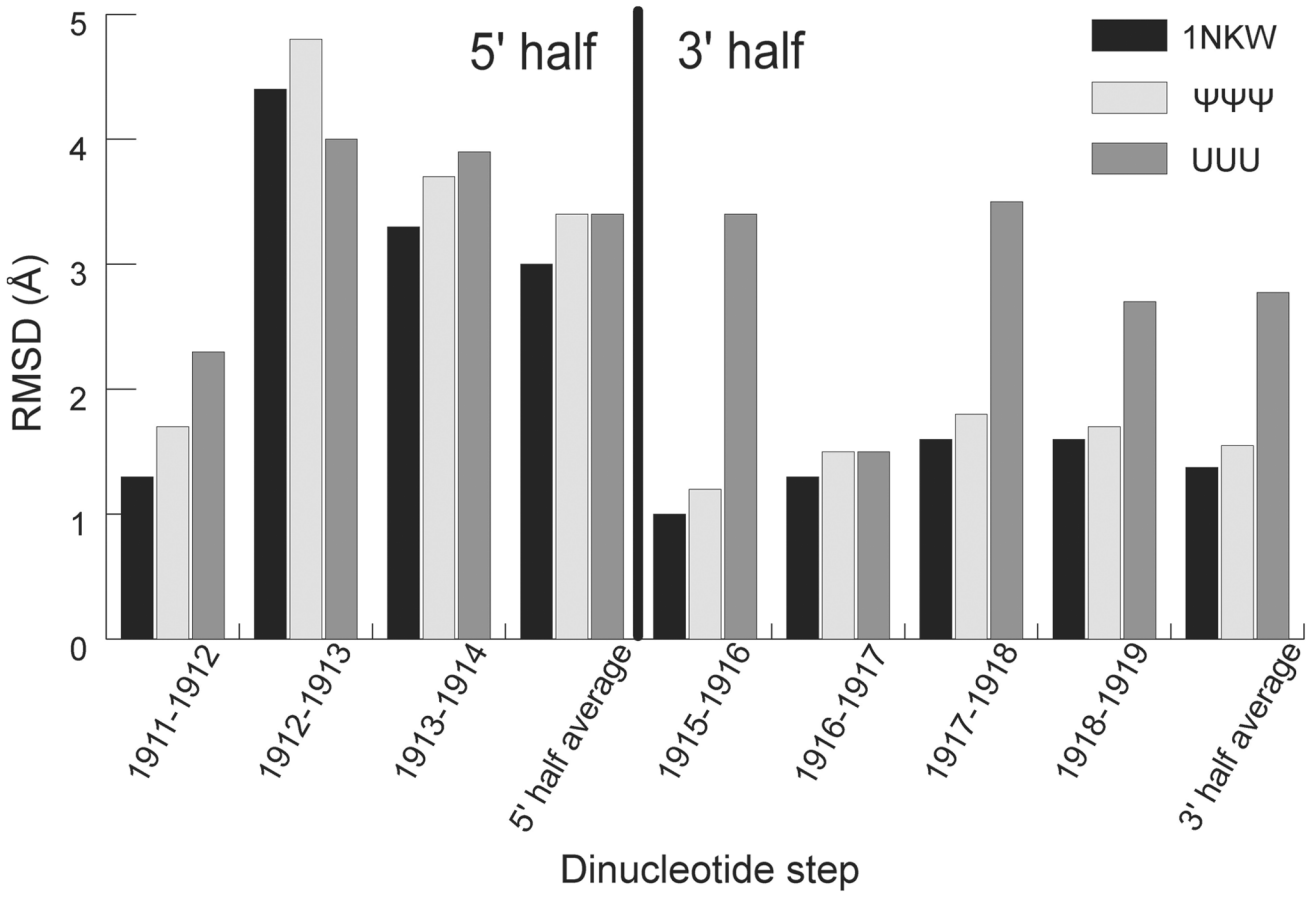
Conformational changes in each dinucleotide step on ribosome assembly are compared. Coordinates of the two neighboring residues in the NMR structures (UUU and ΨΨΨ) and 1NKW were superimposed with corresponding residues in 2I2T and the RMSD for the two residues were calculated with all atoms. Dinucleotide steps were chosen to demonstrate the relative conformational changes within the two residues, when a propagating effect of coordinate translation resulted from other residues, e.g. stem duplex residues, is minimized.

Two of the three Ψ modifications are clustered in the 3' half of the H69 loop, where distinct conformational differences are observed between the NMR structures of UUU and ΨΨΨ (Figures 4c and 5d). In ΨΨΨ, residues Ψ1915, A1916, Ψ1917 and A1918 establish a continuous base-stacking motif, whereas two disruptions in base stacking at the U1915–A1916 and U1917–A1918 steps are observed in the UUU RNA. These conformational differences are also consistent with the chemical-shift changes of the corresponding protons in this region when UUU and ΨΨΨ are compared (Supplementary Figure S3) (54,65). In several other studies, Ψs were shown to have a propensity for base-stacking interactions (6,56,58). The step-wise distances of base mass centers of Ψ1915, A1916, Ψ1917 and A1918 in ΨΨΨ are 4∼5 Å, within the distance range observed for base-stacking interactions in crystal structures (3.5∼6 Å) (59,61). In the NMR structure of UUU, the distances between base mass centers of U1915–A1916 (∼7 Å) and U1917–A1918 (∼9 Å) steps are much larger. Because evidence for a C2'-*endo* sugar pucker of U1915 and U1917 was revealed in the 2D DQF-COSY spectrum of the UUU construct (Supplementary Figure S4a), it is not surprising that these two residues can span much longer distances in UUU compared with the corresponding Ψs in the modified RNA (6,66). Given that the energy of base stacking is approximately proportional to 1/R^6^ (i.e. London dispersion interactions, in which R is the distance between the two base mass centers), the long distances could decrease the stability of the base-stacking interactions between U1915–A1916 and U1917–A1918 in UUU (67). An additional 150 Å^2^ of solvent accessible area from the bases in the Ψ1915 to A1918 region of ΨΨΨ is buried due to base stacking. There is no NMR evidence indicating involvement of the imino groups of Ψ1915 and Ψ1917 in hydrogen-bonding interactions. The conformational rigidity introduced by continuous base stacking promoted by Ψ1915 and Ψ1917 appears on first analysis to be inconsistent with previous thermodynamic studies (37,38), which show that Ψ1915 and Ψ1917 are slightly destabilizing. However, further analysis suggests that stabilization on the 3' part of the loop is compensated by destabilization on the 5' part of the loop.

In previous studies, it was shown that pseudouridylations at 1915 and 1917 exerted a destabilizing effect of 0.8 kcal/mol on the global folding of H69 ΨΨΨ (38), with an individual thermodynamic contribution of 0.7 and 0.3 kcal/mol from Ψ1915 and Ψ1917, respectively (37). The fact that Ψ modifications are able to play opposing thermodynamic roles in the same RNA construct was unanticipated. A rationale for these effects is that bending of the single-stranded region into a loop conformation is more demanding in the stacked environment. Goddard *et al.* reported that an extra 0.5 kcal/mol of enthalpy penalty is paid for each AA step in a DNA hairpin containing a polyA compared with polyT loop. This effect is attributed to base stacking of the purines. In contrast, in a DNA hairpin containing a polyT sequence, whose propensity to form base stacking is low, the thermodynamic penalty is purely entropic (68). The Ψ modifications at 1915 and 1917 appear to behave in a similar manner to the polyA hairpin loop, with Gibbs free energy changes (0.7 and 0.3 kcal/mol for Ψ1915 and Ψ1917) close to the reported value (0.5 kcal/mol•base stacking). This hypothesis is further supported by the fact that no imino protons of Ψ1915 and Ψ1917 participate in hydrogen-bonding interactions, as revealed in both the NMR spectra and the resulting structures, suggesting that the thermodynamic effects are dominated by base stacking. In contrast, in favor of hairpin folding of H69 ΨΨΨ, both N1H and N3H imino protons of Ψ1911 are involved in hydrogen-bonding interactions that stabilize the Ψ1911–A1919 base-pair platform, the terminal residues of the loop.

A continuous base-stacking motif from Ψ1915 to A1918 was observed in crystal structures of the eubacterial ribosomal large subunit before (1NKW, *D. radiodurans*) and after (212T, *E. coli*) being assembled into complete ribosomes (Figure 4e and f) (59,60). Because the sequence and Ψ modifications in H69 from *D. radiodurans* are the same as those in *E. coli*, the crystal structure of H69 from 1NKW is used to represent the structure of H69 from *E. coli* before ribosome assembly, which is not currently available. Comparisons between each dinucleotide step in the H69 loop region of 1NKW and 2I2T indicate that, on assembly of complete ribosomes, residues in the 5' half undergo much larger conformational changes than residues in the 3' half (black bars in Figure 5). Similar trends are observed when the NMR structure of ΨΨΨ and H69 crystal structure from 2I2T are compared (white bars in Figure 5). This observation is not surprising, as all three H69 RNAs (1NKW, 2I2T and ΨΨΨ) contain the conserved Ψ1915 and Ψ1917 residues in the 3' half of the loop region, enhancing local base stacking. Comparison of the NMR structure of UUU and H69 crystal structure from 2I2T reveals large structural differences in both the 5' and 3' halves of the loop region (gray bars in Figure 5), suggesting that more extensive conformational changes are required in H69 on ribosome assembly when Ψ modifications are absent. A continuous base stacking in the 3' half of the H69 loop promoted by Ψ modifications is potentially able to provide consistent structural support, which may play an important role in maintenance of the global conformation during the highly dynamic translation processes (18,21–25).

#### Effects of pseudouridine modifications on the 5' half of H69 loop region

A general A-form RNA conformation is extended into the 5' half (residues from A1912 to C1914) of the loop regions of UUU and ΨΨΨ (Figure 3). One major difference between the crystal structure 1NKW (isolated large ribosomal subunit from *D. radiodurans*) (59) and the NMR structures is that in 1NKW the base moiety of C1914 is stacked on Ψ1915; in contrast in the NMR structures of UUU and >7.6 Å (the distance between A1913H8 and C1914H6) away from the base ring of A1913; in contrast in the NMR structures of UUU and ΨΨΨ the distances between the bases of A1913 and C1914 are much closer (about 4∼5 Å). The proximity of A1913 and C1914 base moieties in space is confirmed by the medium or weak intensity cross-peaks between A1913H8 and C1914H6 in the 2D NOESY spectra of UUU and ΨΨΨ. However, no cross-peaks corresponding to C1914H6 and U/Ψ1915H6 are observed in the same spectra, indicating a breakage of stacking between the bases of C1914 and U/Ψ1915 in the NMR structures (Figure 3), which is also observed in the crystal structure of a ribosome bound to the YaeJ protein (69). The different positioning of the C1914 base in NMR (UUU and ΨΨΨ) and crystal (1NKW and 2I2T) structures suggests that H69 assumes a slightly different conformation in solution, possibly reflecting the promotion of enhanced base stacking of C1914 and Ψ1915 under crystallization conditions. The difference in conditions of the NMR experiments and the crystallization studies (e.g*.* total salt concentration, Mg^2+^ concentration, pH and solution versus crystal state) may also contribute to the differences observed among the NMR structures and the crystal structure (Supplementary Figure S8). Chemical probing studies of whole ribosomes and large subunits also indicated that H69 changes conformation on varying the solution pH and Mg^2+^ concentration (40,62).

Residue C1914 is not the only nucleotide in the 5' half of H69 with conformational flexibility; several reports have indicated that A1913 flips out to form essential interactions with h44 of the 16S rRNA on assembly of the full ribosome to establish the B2a intersubunit bridge (16,59). Factors that can affect the structural dynamics of the 5' half of the H69 loop region may play important biological roles. In summary, modifications at 1911, 1915 and 1917 are capable of altering the conformational behavior of the 5' half of the H69 loop. Even though the global thermodynamic stability of ΨΨΨ is similar to that of UUU (*ΔΔG°_37_* = −0.2 kcal/mol) (70), this zero-sum result possibly elicits a conformational effect in the bridging region between Ψ1911 and the 3' half of the loop containing Ψ1915 and Ψ1917.

## CONCLUSIONS

Helix 69 is a 19-nt stem-loop structure in bacterial 23S rRNAs. The sequence of H69 is highly conserved within each domain of life, and its secondary structure is conserved throughout phylogeny (11). Post-transcriptional modification patterns in H69 also show conservation in all three domains of life (9,10). Even though Ψ modifications in H69 are not essential for the survival and function of bacteria (71), they confer significant advantages to the organism (35,72). Through comparisons of NMR solution structures of UUU and ΨΨΨ, the modifications were shown to alter the structure, dynamics and thermodynamic behavior of the H69 loop (Figure 3).

Pseudouridine modifications in H69 are located in two structural regions. One pseudouridylation is at position 1911, which is co-localized with A1919 at the H69 stem-loop junction. Formation of a canonical Watson–Crick base pair (Ψ1911–A1919), as well as a possible water-mediated hydrogen-bonding interaction involving Ψ1911N1H, helps maintain the stem duplex structure. These interactions allow conformational changes of H69, especially those in the loop region, during the highly dynamic translation process (73). Conformational changes that occur in the H69 ΨΨΨ loop as a result of the presence of the Ψ modifications at positions 1915 and 1917 are restricted to this region, while the stem structure is minimally perturbed (37,38,74). Two additional modifications, Ψ1915 and Ψ1917, occur in the 3' half of the loop region. Significant differences between the NMR structures of H69 UUU and ΨΨΨ are observed in this region. Without Ψ modifications, only A1916 and U1917 participate in base stacking, and there are breaks in the interactions between the U1915–A1916 and U1917–A1918 steps. This local conformation of H69 has not been observed previously in crystal structures of the ribosome, as the RNAs in those structures contained the conserved Ψ modifications. This ‘unstacked’ arrangement likely allows U1915, A1916 and U1917 to be more exposed to solvent and accessible to RluD, the enzyme responsible for converting U to Ψ (34,75). After modifications are incorporated, the arrangement of residues Ψ1915, A1916, Ψ1917 and A1918 is ‘stacked’, in which a continuous base-stacking motif is established. Re-organization of the local conformation on pseudouridylation not only reinforces the structural rigidity in the 3' half of H69 ΨΨΨ loop (76), but also evokes a structural effect that ripples upstream into the 5' half of the loop region to alter local conformational dynamics, which plays an important role in ribosome assembly and maintenance of the ribosomal structural integrity during the translation process.

**Figure 6.**
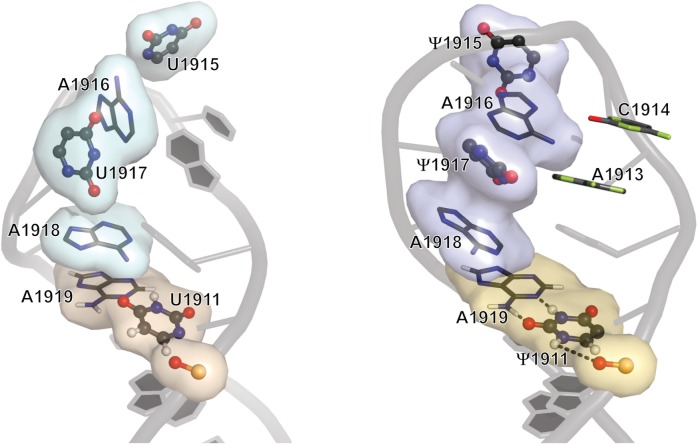
Structural comparisons of the loop regions of H69 UUU (left) and ΨΨΨ (right). Ψ1915 and Ψ1917 alter the loop folding by promoting local base-stacking interactions, and the conformational effects are locked within the loop region by a stabilized Ψ1911–A1919 base pair, together with possible water-mediated hydrogen-bonding interactions involving Ψ1911N1H. The conformational behavior of residues in the 5' half of H69 loop region is affected by pseudouridylation.

In summary, exposure of loop residues U1915, A1916 and U1917 is likely important for substrate recognition and catalysis by RluD. After incorporation of Ψ modifications at positions 1911, 1915 and 1917, these nucleotides appear to work synergistically in modulating the conformation and structural dynamics of the loop (Figure 6), which plays a role in formation and maintenance of bridge B2a during various stages of translation. It has been reported in studies of telomerase (6), spliceosomal (7) and transfer (8) RNAs that Ψ helps pre-structure local RNA motifs for subsequent participation in downstream events; therefore, this work on H69 helps complete the spectrum of roles for Ψ in all major biologically relevant RNA species. The unique structural properties of Ψ and its ability to fine-tune the structure of functionally important RNAs make these sites attractive targets for drug design, and the structures obtained in this work can be applied in the development of novel inhibitors of bacterial ribosome function.

## ACCESSION NUMBERS

The Protein Data bank (PDB) accession numbers of unmodified and modified helix 69 coordinates are 2 meq and 2 mer, respectively. Corresponding accession numbers for chemical shifts and structural restraints in the Biological Magnetic Resonance Bank are 18975 and 18974, respectively.

## SUPPLEMENTARY DATA

Supplementary Data are available at NAR Online.

## FUNDING

National Institutes of Health [GM087596 to C.S.C.]; The purchase of the NMR spectrometer was jointly funded by the Michigan Life Science Corridor [LSC1653], Wayne State Office of the Vice President for Research, College of Science, School of Medicine and the National Institutes of Health [NIH1S10RR016000]. Funding for open access charge: NIH [GM087596].

*Conflict of interest statement*. None declared.

## Supplementary Material

Supplementary Data
