# Supplementary material for: Structure modulation of helix 69 from Escherichia coli 23S ribosomal RNA by pseudouridylations
Source: Nucleic Acids Res. 2013 Dec 26;42(6):3971–81. doi: 10.1093/nar/gkt1329 (PMC3973299; doi:10.1093/nar/gkt1329)
Supplement: Supplementary Data [file supp_42_6_3971__index.html]

Structure modulation of helix 69 from Escherichia coli 23S ribosomal RNA by pseudouridylations — Structure modulation of helix 69 from Escherichia coli 23S ribosomal RNA by pseudouridylations — Supplementary Data 

# Structure modulation of helix 69 from *Escherichia coli* 23S ribosomal RNA by pseudouridylations

## Supplementary Data

files

**Files in this Data Supplement:**

- Supplementary Data - pdf file
