# Supplementary material for: Structure modulation of helix 69 from Escherichia coli 23S ribosomal RNA by pseudouridylations
Source: Nucleic Acids Res. 2013 Dec 26;42(6):3971–81. doi: 10.1093/nar/gkt1329 (PMC3973299; doi:10.1093/nar/gkt1329)
Supplement: Supplementary Data [file supp_gkt1329_nar-02993-r-2013-File008.pdf]

Supplementary Data for

Structure Modulation of Helix 69 from *Escherichia coli* 23S Ribosomal RNA by Pseudouridylations

Jun Jiang, Raviprasad Aduri[#], Christine S. Chow, and John SantaLucia, Jr.*

Department of Chemistry, Wayne State University, Detroit, MI, 48202, USA

[#]Current address: Department of Biological Sciences, BITS Pilani K K Birla Goa Campus, Goa, 403726, India.

*Corresponding author: jsl@chem.wayne.edu

Table of contents:

Supplementary Figure S1. 2D NOESY spectrum of ΨΨΨ shows the cross peaks of Ψ1911N3H-A1919H2 and Ψ1911N1H-H6 2

Supplementary Figure S2. The base H8/6 – sugar H1' regions and the sequential connectivity patterns in the 2D NOESY of UUU and ΨΨΨ are compared. 3

Supplementary Figure S3. Resonance shifts of the base protons and sugar H1' between UUU and ΨΨΨ are given. 4

Supplementary Figure S4. 2D DQF-COSY spectra of UUU H1'-H2' region and ΨΨΨ Ψ1915 H6-H1' region are shown... 5

Supplementary Figure S5. G1907-U1923 wobble pairs and their positions relative to G1922 in UUU and ΨΨΨ are shown. 6

Supplementary Figure S6. Ψ1911N1H is exposed to the solvent. 7

Supplementary Figure S7. Conformations of Ψ1911 in the NMR structure of ΨΨΨ and crystal structures 1NKW and 2I2T are compared. 8

Supplementary Figure S8. Global fitting of the H69 NMR structures with the electron density map of H69 in crystal structure 2I2T 9

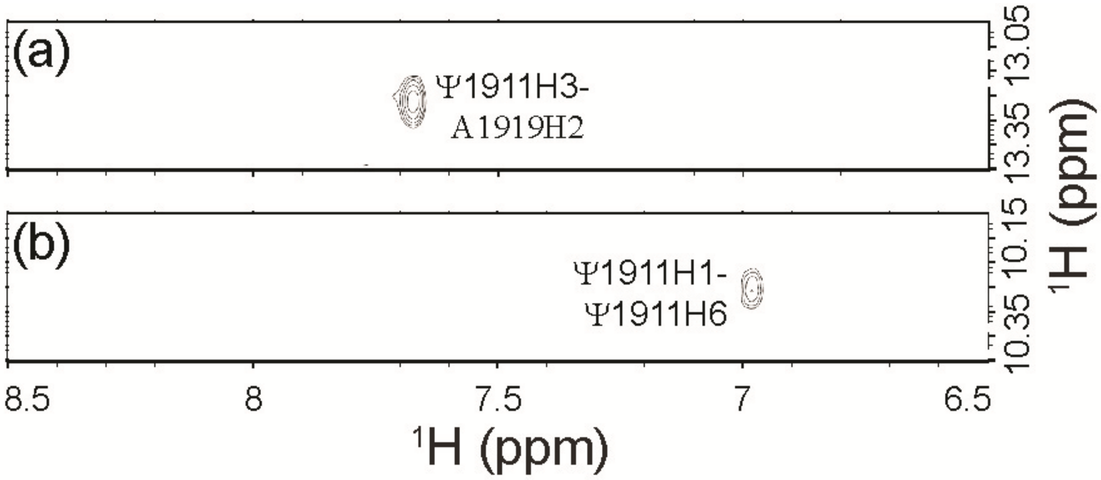

Supplementary Figure S1. 2D NOESY spectrum of ΨΨΨ shows the cross peaks of Ψ1911N3H-A1919H2 (a) and Ψ1911N1H-H6 (b). The resonances of the imino protons are assigned. Chemical shifts of the imino protons in (a) and (b) agree with those observed in the 2D NOESY spectra of UUU and ΨΨΨ (H_2O/D_2O 90%/10%) (Figure 2).

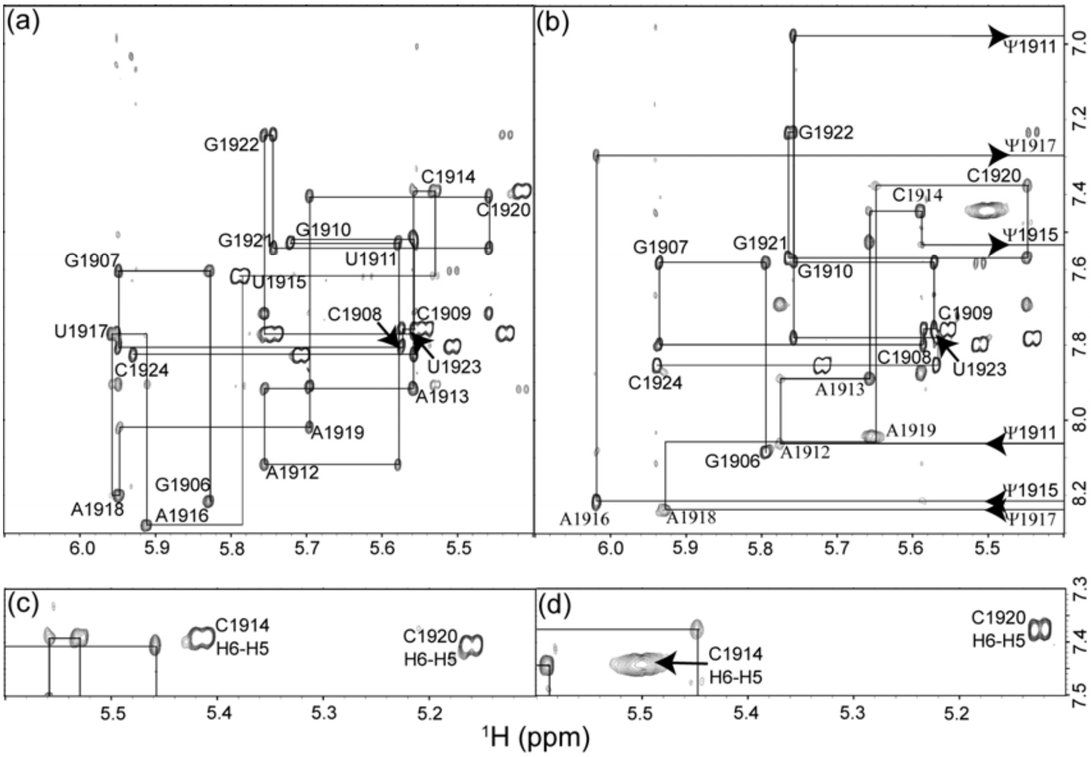

Supplementary Figure S2. The base H8/6 – sugar H1' regions and the sequential connectivity patterns in the 2D NOESY of UUU (a) and ΨΨΨ (b) are compared. The H1' resonances of Ψ1911, Ψ1915, and Ψ1917 in ΨΨΨ are shifted to 4.672, 4.673, and 4.624 ppm, respectively. An upfield shift of the H1' resonance is a signature of Ψ modification. Compared to the H6-H5 cross peak of C1914 in UUU (c), the line-width of the corresponding cross peak in ΨΨΨ (d) is significantly broadened, while the C1920 H6-H5 cross peaks in the two spectra do not show such a difference.

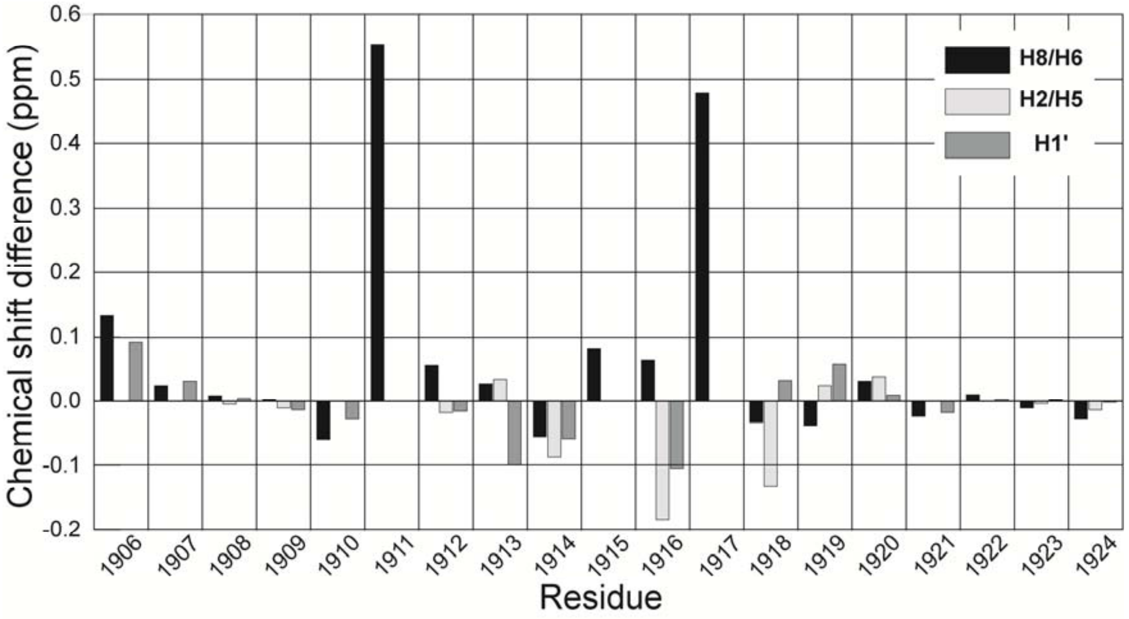

Supplementary Figure S3. Resonance shifts of the base protons and sugar H1' between UUU and ΨΨΨ are given. Resonance shifts of sugar H1' involved in Ψ modifications (1911, 1915, and 1917) are not shown for clear illustration of the shifts with a smaller scale.

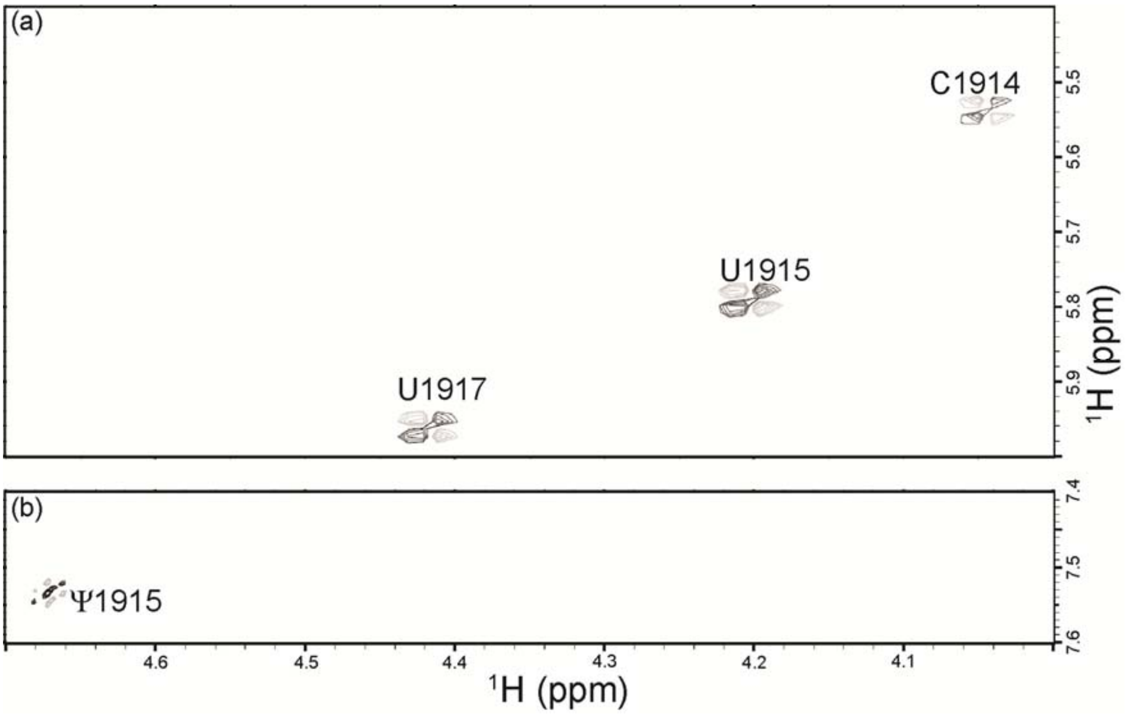

Supplementary Figure S4. 2D DQF-COSY spectra of UUU H1'-H2' region (a) and ΨΨΨ Ψ1915 H6-H1' (b) are shown. Intense cross peaks of H1'-H2' from C1914, U1915, and U1917 are observed in (a), indicating that the ribose moieties in these three residues assume a *C2'-endo* sugar pucker conformation. A four-bond weak cross peak of Ψ1915 H6-H1' is observed in (b), which suggests a co-planar geometry of all the atoms involved in the magnetization transfer (H6-C6-C5-C1'-H1').

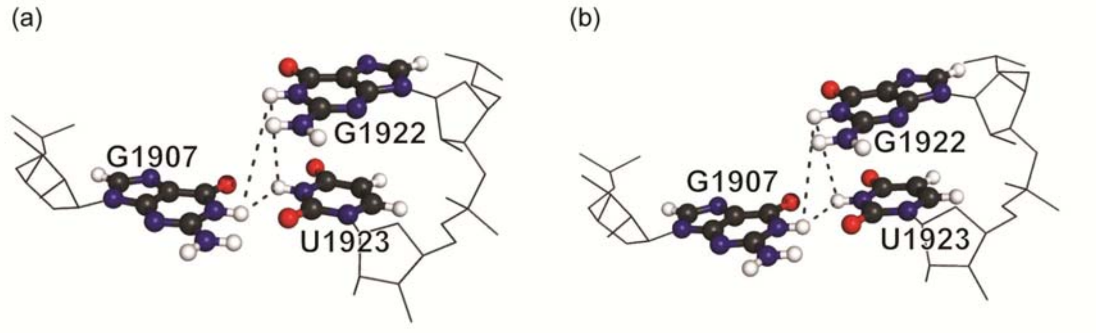

Supplementary Figure S5. G1907-U1923 wobble pairs and their positions relative to G1922 in UUU (a) and ΨΨΨ (b) are shown.

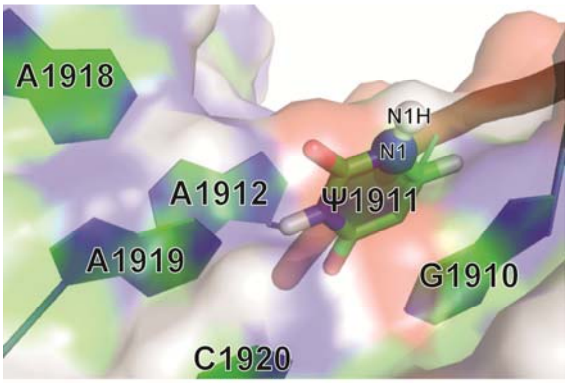

Supplementary Figure S6. Ψ1911N1H is exposed to the solvent, and there is sufficient space to fit a water molecule between Ψ1911N1H and Ψ1911O2P (see Supplementary Figure S7).

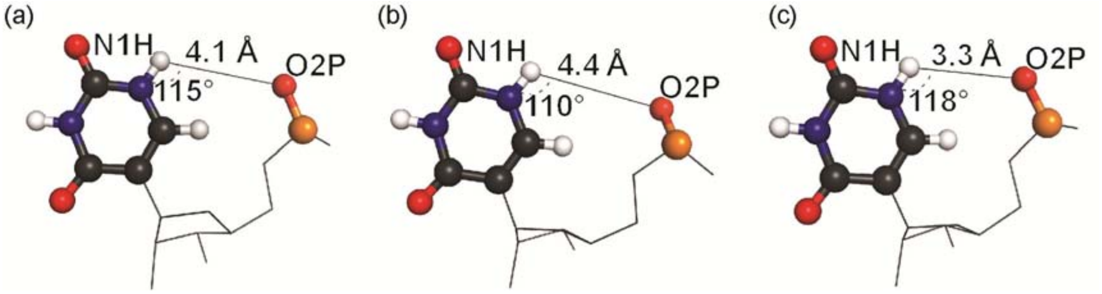

Supplementary Figure S7. Conformations of Ψ1911 in the NMR structure of ΨΨΨ (a) and crystal structures 1NKW (b) and 2I2T (c) are compared. The atoms of N1H and O2P are highlighted in the structures. The geometries of Ψ1911 are similar in all three structures.

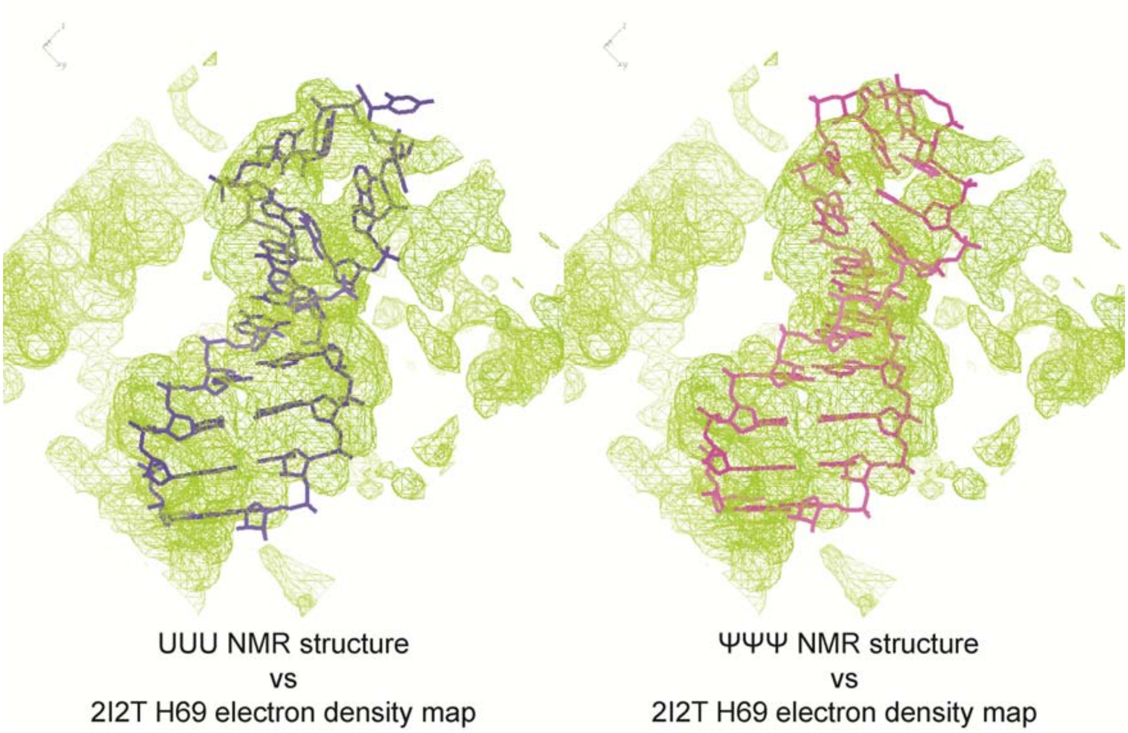

Supplementary Figure S8. Global fitting of the H69 NMR structures with the electron density map of H69 in crystal structure 2I2T. H69 assumes a stem-loop structure in the NMR structures similar to that in the crystal structure 2I2T. Residues in the loop region of the NMR structures can't be fitted into the electron density map of H69 in crystal structure 2I2T. The electron density map was "fetched" and visualized with Coot (1). The coordinates of the NMR structures were aligned with the coordinates of H69 in crystal structure 2I2T by "LSQ superpose" function.

77. Emsley, P. and Cowtan, K. (2004) Coot: model-building tools for molecular graphics. *Acta Crystallogr. D. Biol. Crystallogr.*, **60**, 2126-2132.
